# Fractured Neck of Femur Management at a District General Hospital: Adherence to NICE guidelines CG124 for total hip replacement

**DOI:** 10.1051/sicotj/2020045

**Published:** 2020-12-04

**Authors:** Srikanth Mudiganty, Ilias Kosmidis, John Edwin

**Affiliations:** 1 Senior Clinical Fellow, Trauma and Orthopaedics, East Lancashire Hospitals NHS Trust Haslingden Road BB2 3HH Blackburn Lancashire United Kingdom; 2 Consultant, Trauma and Orthopaedics, Basildon and Thurrock University Hospital Nethermayne SS16 5NL Basildon Essex United Kingdom

**Keywords:** Hip fracture, Arthroplasty, NICE

## Abstract

*Introduction*: The National Institute for Health and Care Excellence (NICE) in 2011 declared standards in the management of fracture neck of femur (NOF) patients suggesting a total hip replacement (THR) if necessary criteria were met. The Best Practice Tariff (BPT) states all NOF fracture patients should be operated on within 36 h of presentation to Accident & Emergency. We conducted this retrospective study for the years 2016–2018 to evaluate the adherence to these guidelines by Basildon and Thurrock University Hospital and compared the results with national standards. *Methods*: Data for the period from 2016 to 2018 was collected from the National Hip Fracture Database (NHFD) retrospectively. The data was analysed to calculate various procedures performed for fracture NOF fixations, the number of THR’s for displaced intracapsular fracture NOF, and percentage of patients operated within 36 h and evaluated reasons for the delay. *Results*: Over the 3 years, the number of THR eligible displaced intracapsular neck of femur fracture patients that underwent THR was above the national average. Across all 3 years, the number of patients who underwent surgery within 36 h was less than the national average. Administrative/logistic reasons for the delay were the major cause for delayed surgery in all 3 years. *Conclusion*: Compliance with the NICE guidelines and achievement of national standards in NOF fracture care is achievable by most district general hospitals. Awareness and implementation of NICE guidelines for THRs need to be enhanced. A sustained, continual team effort and strict vigilance are necessary to prevent delayed surgery.

## Introduction

The neck of femur (NOF) fractures represents an increasing volume of patients presenting to orthopaedic departments, with over 100,000 admissions predicted in 2020 at an estimated cost of greater than £2 billion. With the high incidence of the condition, there has been a systemic shift toward improved compliance with evidence-based best practice management. In 2011, the National Institute of Clinical Excellence (NICE) issued guidelines for the management of hip fractures in adults, with recommendations spanning pre-operative management through to discharge and follow-up [1]. These recommendations followed the introduction of the fragility hip fracture best practice tariff (BPT) in 2010, which saw a reduction in the financial compensation paid to hospitals for the management of hip fractures in adults with additional funding provided if hospital trusts met defined targets [2]. These targets include prompt surgery (within 36 h) from arrival in an emergency department, (or time of diagnosis if an inpatient); admission under the joint care of a consultant geriatrician and a consultant orthopaedic surgeon, with assessment by a geriatrician in the preoperative period; postoperative geriatrician-directed multi-professional rehabilitation team; and secondary prevention fracture assessments (falls and bone health).

NICE guidelines provide clear recommendations regarding the surgical management of fractured NOF. These state that patients with a displaced intracapsular NOF fracture should be received either a total hip replacement (THR) or hemiarthroplasty depending on their pre-fall status. THR should be offered to patients who are able to walk independently outdoors with no more than the use of a stick, who have no cognitive impairment (an Abbreviated Mental Test Score of 8 or more at admission) and who are deemed medically fit for anaesthesia and the procedure (American Society of Anaesthesiology Grade of no more than 2).

In this study, we sought to determine if a busy, district general hospital (DGH) trust can adhere to NICE guidelines and offer THRs to eligible patients. In addition, we identified the reasons for delay in surgery, and the number of patients who were eligible for THR but offered hemiarthroplasty. These figures were then compared to the national average.

## Methods

A retrospective review of data collected over a three-year period from 2016 to 2018 was collected from the NHFD for all patients who had their surgery performed in Basildon and Thurrock University Hospital. The following parameters were analysed: type of surgery, time to surgery, ASA grade, AMTS documented at admission, and pre-fall mobility status. If a patient’s surgery was delayed by >36 h from admission, their clinical records were analysed in order to elucidate the cause of the delay.

## Results

**Figure 1:**
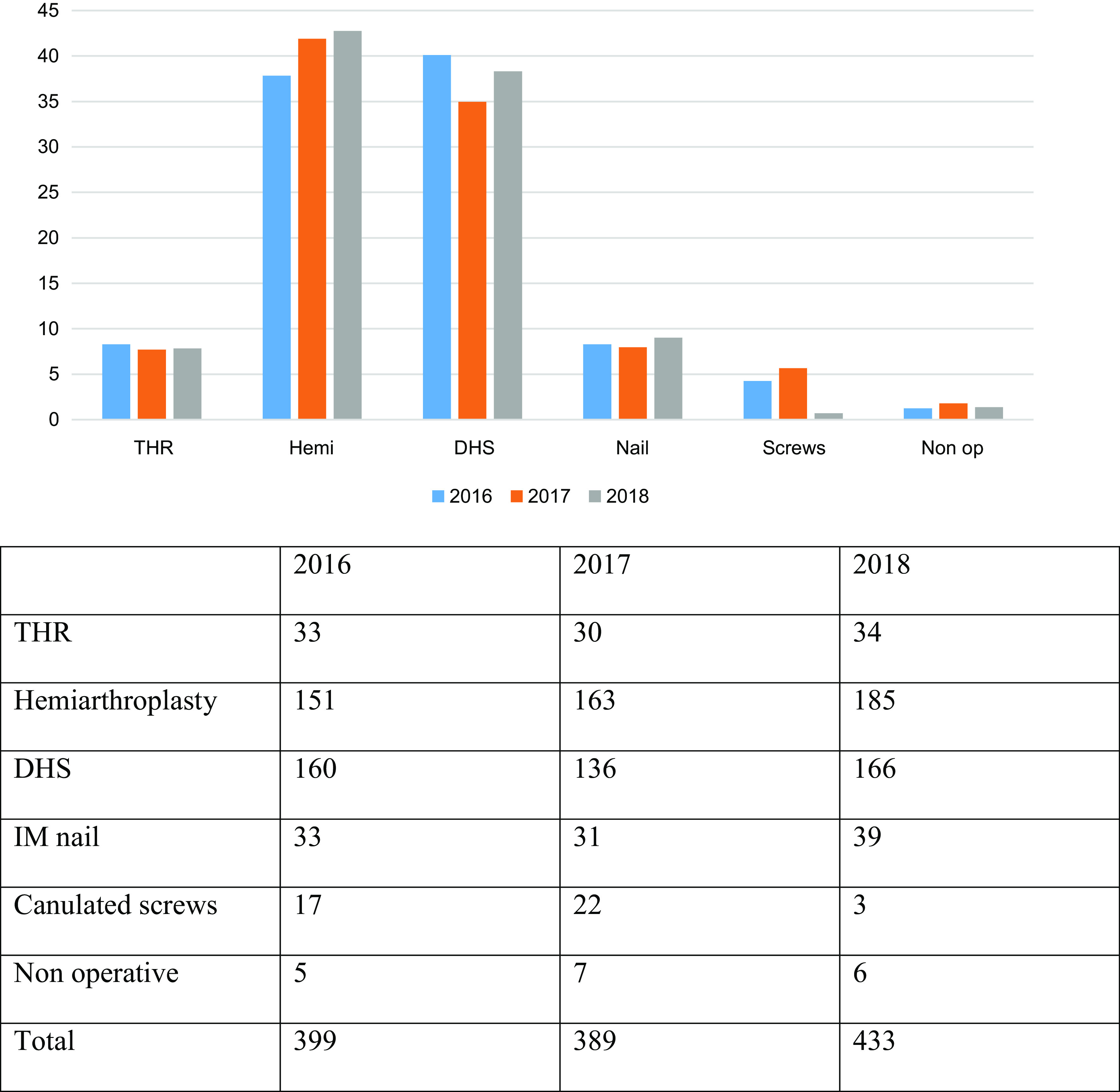
Various NOF fracture treatments (%).

Over the three-year period, 1221 patients presented with a fractured NOF (Figure 1).

In 2016, there were 399 NOF fractures, 33 of which were treated with a THR. 151 were treated with a hemiarthroplasty. These findings are similar for 2017 in which 30 were managed with a THR and 163 were hemiarthroplasty. Similarly, in 2018 these figures were 34 and 185 for THR and hemiarthroplasty respectively.

**Figure 2:**
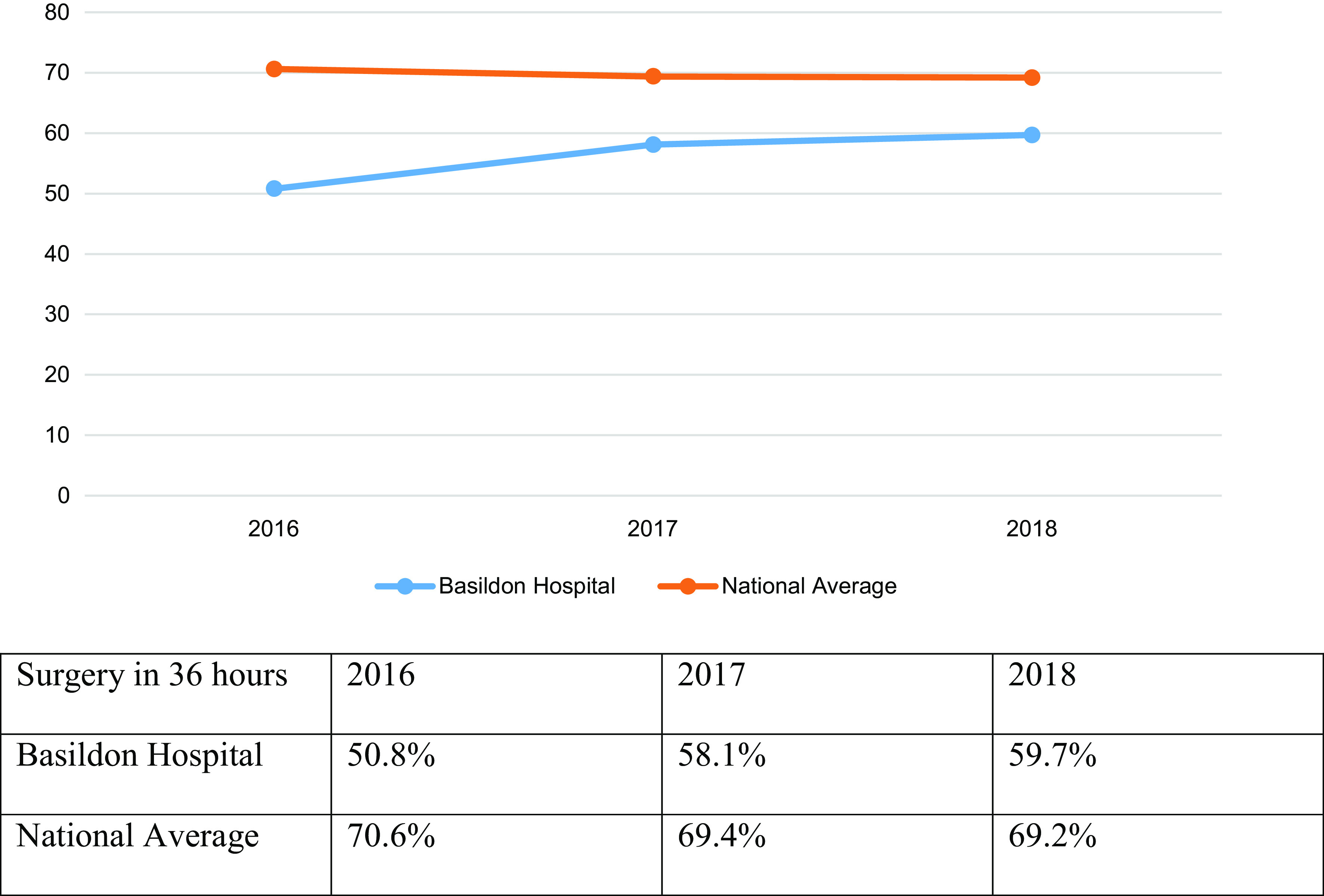
Surgery within 36 h (% of cases).

In 2016, 50.8% of patients with NOF fracture achieved the BPT of surgery within 36 h, compared to 58.1% in 2017 and 59.7% in 2018. These figures were below the national average (Figure 2).

**Figure 3:**
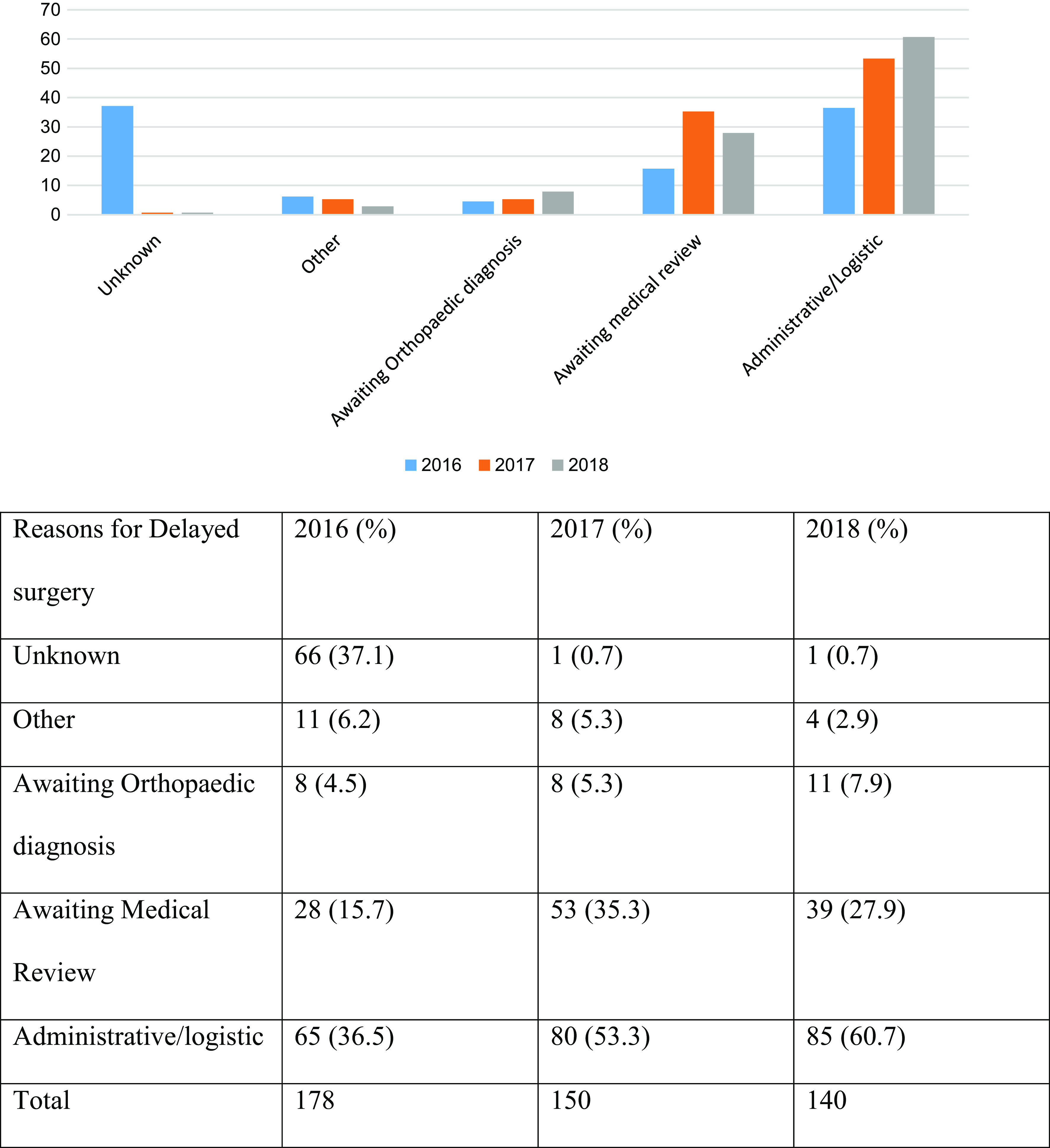
Reasons for delay in surgery.

The most common reason for delayed surgery was classified as unknown in 2016, administrative/logistic in 2017 and 2018. However, there was no significant difference between hemiarthroplasty and THR in terms of delayed surgery (Figure 3).

**Figure 4:**
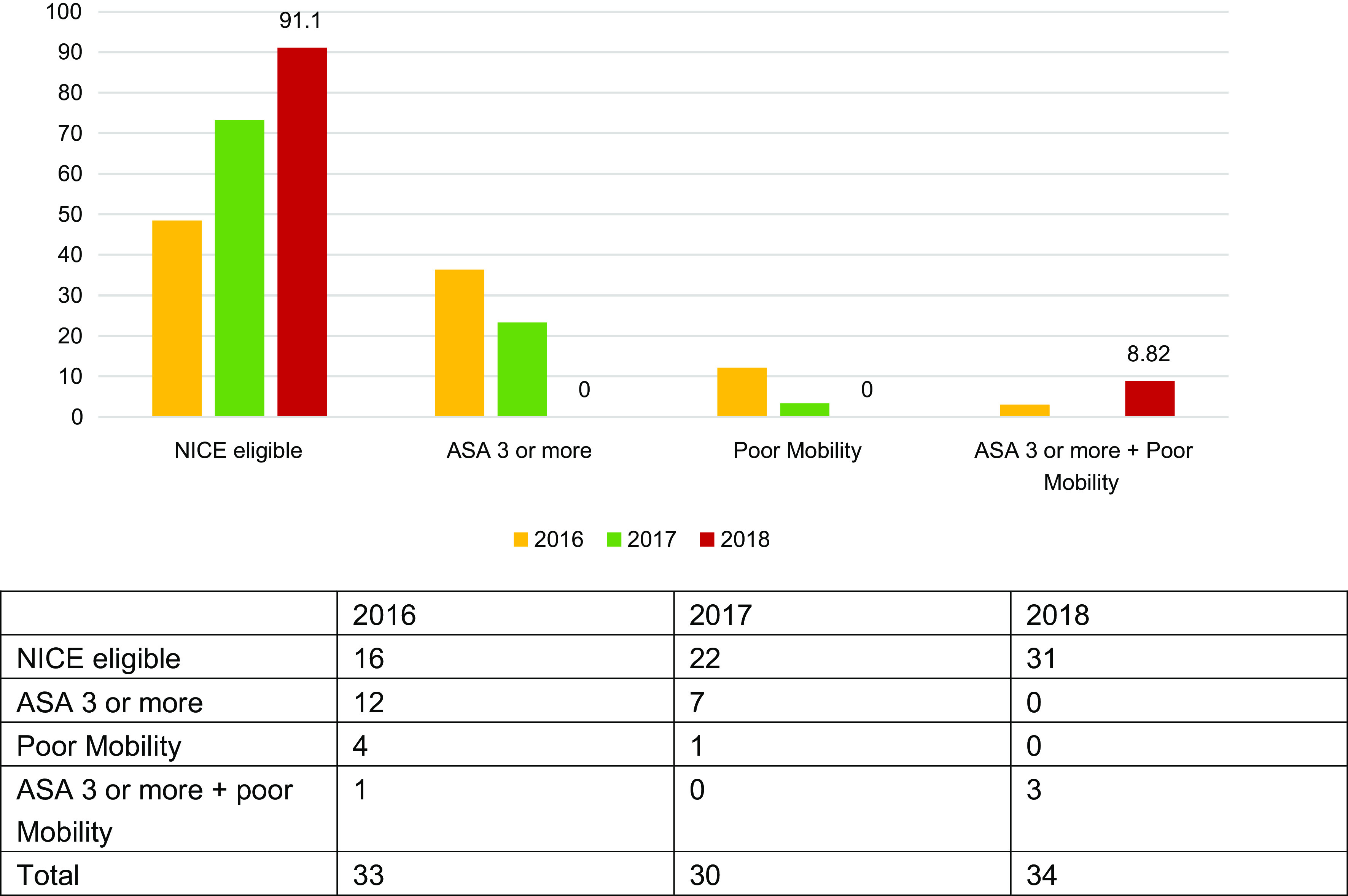
THR patients (%) who fulfilled NICE criteria and reasons for those who did not.

Of patients who underwent THR, 16 (48.5%) in 2016, 22 (73.3%) in 2017, and 31 (91%) in 2018 were deemed eligible for a THR by NICE guidelines. However, out of the total number of THR eligible displaced intracapsular fracture neck of femur patients, 59.3% (2016), 45.8% (2017), and 83.8% (2018) actually underwent a THR. These are the patients who were ASA 2 or less, AMTS 8 or more, and walked independently or with a single aid outdoors. The hospital outperformed the national standards in this parameter (Figure 4).

In total, 496 patients received a hemiarthroplasty across the three-year period. In 2016, 11 fulfilled NICE guidelines for a THR. Similarly, in 2017, 26 who received a hemiarthroplasty would have been eligible for a THR, and 6 in 2018 were also deemed eligible (Table 1).

**Table 1:** Percentage of THR eligible patients who received a THR.

	2016	2017	2018
Patients who met NICE criteria for THR and underwent hemiarthroplasty	11	26	6
Patients who met NICE criteria for THR and underwent THR	16	22	31
Total number of patients who met NICE criteria for THR	27	48	37
% of THR eligible patients who received THR	59.3	45.8	83.8
National average	30.4	31.4	33.2

## Discussion

Over the study period of 3 years, there a substantial rise in the number of THR’s performed on eligible patients. These numbers were consistently higher than the national average [3–5]. This demonstrates an increased awareness among the NOF fracture team members with regard to the NICE guidelines and BPT rules. Perhaps, this could be attributed to our audit findings and their discussion in the audit meeting. In spite of the consistent rise in the number of eligible THRs, there were patients who underwent THR even though they did not satisfy the criteria outlined in the NICE guidelines. The most common reason being THR performed on patients deemed was ASA grade 3 and above. Although, NICE does mention fitness for surgery as an eligibility criterion for THR, for quantifying purposes this is presumed as ASA grade 2 or below. In contrast, a proportion of patients who were labelled ASA grade 2 and eligible for THR as per the NICE guidelines underwent hemiarthroplasty. A possible reason for this is that the ASA grading is ambiguous and varies with different Anaesthetists and the surgeons are not correctly schooled in determining the ASA grade [6]. As in many other NHS Hospitals, in Basildon Hospital the Anaesthetists are not involved in the admission phase of the NOF patients and the fitness for THR is determined by the surgeon. Although, this is discussed in the pre-op WHO checklist meeting, perhaps early involvement of the anaesthetist may lead to improved outcomes.

The number of patients who were operated on within 36 h as outlined in the BPT rules showed a steady increase over the 3-year period. However, Basildon Hospital lagged behind the national average in all 3 years. In 2016, the most common reason for delayed surgery was identified as unknown being attributed to poor documentation, followed by Administrative/logistic reasons as per NHFD. In the next 2 years, the most common reason for delayed surgery was administrative/logistic such as lack of theatre space, equipment, and/or trained staff. It needs to be highlighted that for a NOF fracture patient to be operated in 36 h of presentation requires a coordinated team effort. It starts with the patient being seen in A&E, Trauma & Orthopaedic (T&O) on-call team, ward team, orthogeriatric check, pre-op optimisation, Anaesthetic team review and surgeon assessment and theatre team preparedness. At the same time, the availability of theatre space is an issue especially in busy DGH like ours which needs conscientious management. Our DGH has a robust clinical governance structure lead by a NOF T&O lead, Orthogeriatric consultant, NOF nurse, and other health care personnel who lead, supervise and maintain appropriate standards.

In our study, there was no significant delay in the time for theatre between THR and hemiarthroplasty suggesting the availability of qualified arthroplasty surgeons the majority of the time. Indeed, Basildon Hospital has 8 qualified arthroplasty consultants capable of performing THRs. However, improvement is needed in the selection of patients undergoing THRs and NICE guidelines should have adhered in this surgical decision.

## Conclusions

This retrospective study highlights the fact that a busy, medium-sized district general hospital can deliver neck of femur fracture care at par with national standards in terms of performing THRs for eligible neck of femur fracture patients in compliance with NICE guidelines. There was a percentage of patients who did not undergo a THR in spite of satisfying the NICE criteria. This could possibly lead to an inferior outcome in these patients. Increased awareness among the surgeons, Anaesthetists regarding the NICE guidelines and availability of surgeons capable of performing THRs is something that needs to improve upon to achieve better results.

There is ample scope for improvement in terms of the timing of surgery in line with BPT rules. Early involvement of the Ortho-geriatrician and Anaesthetist would help prevent delayed surgery, especially in medically unwell patients. Administrative/logistic reasons for delayed surgery are preventable to a major extent and need team effort and coordination. There is no significant difference in time to surgery between hemiarthroplasty and THR indicating the availability of surgeons to perform THRs. The reason being that in our department, 8 consultants are competent to perform a THR. However, we do appreciate that this may not be the case in every hospital. The neck of femur fracture patient care should be audited in accordance with the NICE guidelines and audit findings should be evaluated to improve patient care.
